# Attrition from Care and Barriers to PrEP Use Among Key Populations in Kinshasa, DRC: A Multiple Methods Study

**DOI:** 10.1007/s10461-025-04809-5

**Published:** 2025-07-07

**Authors:** Natalia Zotova, Alisho Shongo, Patricia Lelo, Nana Mbonze, Didine Kaba, Paul Ntangu, Qiuhu Shi, Adebola Adedimeji, Kathryn Anastos, Marcel Yotebieng, Viraj Patel, Jonathan Ross

**Affiliations:** 1 Division of General Internal Medicine, Department of Medicine, Albert Einstein College of Medicine, 3300 Kossuth Ave, Bronx, NY 10467 USA; 2 School of Public Health, University of Kinshasa, Kinshasa, Democratic Republic of Congo; 3 Kalembelembe Pediatric Hospital, Kinshasa, Democratic Republic of Congo; 4 National AIDS Control Program (PNLS), Provincial Coordination, Kinshasa, Democratic Republic of Congo; 5 New York Medical College, Valhalla, NY USA; 6 Department of Social Sciences and Health Policy, Wake Forest University School of Medicine, Winston-Salem, NC USA

## Abstract

Female sex workers (FSW) and gay and other men who have sex with men (MSM) are disproportionately affected by HIV. Oral pre-exposure prophylaxis (PrEP) is increasingly available in African countries, including the Democratic Republic of Congo (DRC), but data on factors influencing PrEP use remain limited. This multiple methods study examined PrEP attrition patterns and barriers to engagement among FSW and MSM in Kinshasa, DRC, using programmatic data from five sites, clinical records, and qualitative interviews. Logistic regression identified factors associated with attrition; qualitative data were thematically analyzed. Among 8,822 FSW and MSM eligible for PrEP in 2019–2021, only 24% (*n* = 2,070) initiated it. Of 809 FSW initiators, 33% (*n* = 268) were lost to follow-up by 1 month and 78% (*n* = 421) by 3 months. Among 1,261 MSM, 26% (*n* = 332) and 87% (*n* = 808) were lost by 1 and 3 months, respectively. For FSW, prior PrEP use and recent STIs were associated with higher attrition at 1 month. Older age, more sexual partners, income beyond sex work, and no prior PrEP use were linked to lower attrition at 3 months. Among MSM, no prior PrEP use predicted higher attrition at 1 month but lower attrition at 3 months. Qualitative findings identified stigma, side effects, dislike of daily dosing, and limited services for key populations at risk of HIV as major barriers. Findings underscore the need for improved PrEP messaging, including information on side effects decreasing over time. Raising awareness among key and general populations may reduce stigma and improve PrEP engagement.

## Introduction

HIV remains a major public health challenge, disproportionately affecting sub-Saharan Africa (SSA). Of the estimated 39 million people living with HIV (PLWH), 25.6 million reside in the SSA region. In 2022, of the 510,000 new HIV infections reported in SSA, an estimated 25% occurred among key populations (KPs) —including female sex workers, gay men and other men who have sex with men—as well as their sexual partners and clients [1].

Pre-exposure prophylaxis (PrEP) is highly effective in reducing the risk of HIV acquisition [2, 3]. Oral PrEP has become increasingly available in low- and middle-income countries, including SSA. It was first introduced in South Africa in 2016, with initial provision at demonstration sites, followed by a scaled-up provision for KPs [4]. National programs to deliver PrEP to population groups at high risk of HIV, including female sex workers (FSW) and men who have sex with men (MSM), are rapidly scaling up in other African countries, including the Democratic Republic of Congo (DRC). Early demonstration studies among KPs in Benin [5], Kenya and Uganda [6], Senegal [7], and South Africa [8] have shown PrEP acceptability and effectiveness. However, PrEP uptake and retention among FSW and MSM in real-world settings remain suboptimal [9–16].

The HIV epidemic in Western and Central Africa differs from that in Eastern and Southern Africa. While HIV prevalence among the general population in Western and Central Africa continues to fall, hovering around 1% or lower, FSW and MSM are disproportionately affected by HIV [17–19]. Understanding HIV prevention use and needs among KPs is essential to inform ongoing efforts to address the epidemic. Yet, few data on the PrEP continuum of care have been reported from this region. Evidence from Cameroon pointed to low rates of PrEP initiation and elevated discontinuation among FSW and MSM: 45% of eligible individuals had initiated PrEP, and 37% and 19% of clients who initiated PrEP continued using it at 3 and 12 months, respectively [18]. A study from Rwanda found similar uptake but higher continuation rates: PrEP uptake was 46% among FSW and 35% among MSM. Among PrEP initiators, 79% of FSW and 88% of MSM continued using it at 12 months [17]. Another study from Rwanda found that 77% of FSW and MSM combined continued using PrEP at 12 months post-initiation [19].

The DRC, the largest country in sub-Saharan Africa by land mass, has been scaling up PrEP since 2019, focusing on key population groups at risk of HIV [20–23]. An early demonstration study conducted in 2018 reported high acceptability of PrEP [21, 22]. Despite an estimated 60,000 individuals having initiated PrEP in the DRC since 2019 [24], no subsequent research has examined patterns of PrEP use among FSW and MSM, barriers to PrEP use or the experiences of PrEP users. This multiple methods study examined attrition from care among Congolese FSW and MSM who were eligible for PrEP and sought to understand the underlying factors of PrEP engagement in a real-world setting.

## Methods

### Study Design, Population, and Setting

This multiple-methods study collected and analyzed data from three sources: programmatic data from five participating healthcare facilities and drop-in centers (DICs); quantitative data from clinical registries of FSW and MSM PrEP users; and in-depth interviews with FSW and MSM. The study was approved by the Institutional Review Board (IRB) of Albert Einstein College of Medicine (protocol #2020–12619) and the Ethical Committee of the University of Kinshasa School of Public Health (protocol #ESP/CE/110/2021).

The DRC is located in Central Africa and has a population of over 100 million. HIV prevalence among the adult population is approximately 1% [25], but is two or more times higher in the capital city of Kinshasa, which has a population of 17 million [26], and disproportionately higher among FSW (7.5%) and MSM (7.1%) [25]. National guidelines recommend daily oral PrEP for individuals at high risk of HIV, including FSW, MSM, transgender people, persons who inject drugs, and HIV-negative partners of people living with HIV.

To enhance the involvement of key populations at risk in HIV services, the DRC Ministry of Public Health designated certain health facilities and DICs as “KP-friendly” centers. In the context of widespread marginalization and stigmatization of LGBTQ + individuals and sex workers, KP clients are offered a range of services in low-stigma, safe spaces. These services include outreach, peer education, testing, HIV and STI prevention and treatment, as well as free access to PrEP [21]. The U.S. President’s Emergency Plan for AIDS Relief (PEPFAR) partners with the DRC Ministry of Health and the National Program for HIV to provide PrEP at designated facilities. We selected five of these KP-friendly sites for the study, as they were widely preferred by KPs and were the most active in PrEP distribution at the time of data collection in 2022. These sites included four community-based, nongovernmental organizations serving KPs—primarily MSM and FSW—as well as one medical center offering HIV and STI prevention and care services to KPs, among other health services. Although the five study sites varied in size, they each consulted, on average, over 1,000 KP clients annually at the time of the study.

The DRC’s national HIV prevention guidelines state that PrEP is provided to adults at substantial risk of HIV who are HIV negative, have normal renal function, and who are willing to take a daily medication. During an initial visit to a health facility or a DIC—often as part of an STI assessment or treatment—health care providers or community health workers share information about PrEP and assess whether individuals are at high risk of HIV acquisition and eligible for PrEP. Individuals on PrEP receive clinical consultations, medication refills, and creatinine testing every three months.

### Data Sources

We utilized data from three sources. First, programmatic data was obtained directly from staff at the five participating sites. This included aggregate numbers of PrEP assessments, prescriptions, and refills, collected quarterly from all sites. Second, the research team extracted routine clinical data from health center registries of FSW and MSM who initiated PrEP between 2019 and 2022 at these same five sites. Third, we conducted individual in-depth qualitative interviews (IDIs) with FSW and MSM at the participating sites.

### Data Collection

To obtain programmatic data, we directly queried administrators or staff at each participating site. They used their routinely collected information to provide aggregate, de-identified data on the number of individuals who were assessed, deemed eligible, and initiated on PrEP at each site from 2019 to 2021. This was the first time that these primary data on the PrEP services cascade were collected in the DRC. We did not assess the validity of these data.

To form a retrospective cohort of FSW and MSM using PrEP, research staff used clinical registries to extract individual-level data on individuals who initiated PrEP from 2019 to 2021, including sociodemographic information, HIV risk, and sexual health. These data were then stripped of identifiers and entered into REDCap, an electronic research data capture database. Repeat subjects—those who re-initiated PrEP on multiple occasions—were not included in the dataset. The IRB of the Albert Einstein College of Medicine and the Ethics Committee of the Kinshasa School of Public Health waived the requirement for informed consent for the collection and analysis of deidentified data from this retrospective cohort.

Data from clinical registries were most complete for questions related to sociodemographic information, sexual behavior (e.g., number of sexual partners in the past six months, condom use), sexual health (e.g., STIs in the past six months), and previous use of PrEP among FSW and MSM. Responses to questions regarding reported STI symptoms, injection drug use, and sexual contact with individuals at high risk of HIV (e.g., people living with HIV and people who inject drugs) were almost universally negative. We assumed that collecting detailed responses to these questions was not a primary focus for providers or community health workers during the completion of PrEP clinical registries. Therefore, we chose not to include these variables in our statistical models.

Given that this study used deidentified data, information on the geographic distribution of subjects was limited to the names of Kinshasa health zones (HZs). A primary unit of health care organization in the DRC, there are 519 HZs in total—35 of which are in Kinshasa. Each HZ serves a large population and can have a significant catchment area.

Criterion purposive sampling was used to recruit participants for semi-structured in-depth interviews (IDIs) from the retrospective cohort, based on key criteria such as key population group (FSW and MSM) and PrEP use status (currently using or stopped using). To include diverse perspectives on PrEP, we also used clinical registries at participating sites to identify FSW and MSM who were evaluated for but never initiated on PrEP, and invited them to participate.

A verbal script was used during the initial contact with prospective participants, which specified that we were contacting them because they had been seen at participating sites in the previous three years and might benefit from a medical treatment called PrEP. The script provided brief information about the study and emphasized that participation was voluntary. In-person IDIs were then scheduled with interested individuals, who signed a written informed consent prior to the beginning of the interview.

The IDI sample size was guided by the concept of thematic data saturation, which is generally achieved in 20 to 30 qualitative interviews [27, 28]. In our study, we decided a priori on a sample size of *N* = 30 to capture a range of participants’ experiences and reach thematic saturation. From November 2021 through March 2022, trained research staff conducted 30 IDIs in French and Lingala to collect information about participants’ awareness of and willingness to use PrEP, experiences with PrEP, and barriers to and facilitators of PrEP use. A short questionnaire was administered at the end of the IDIs to collect sociodemographic and health behavior information. The IDIs were conducted in private rooms at participating sites and were audio-recorded. Participants received 10,000 Congolese Francs (approximately 5 USD) to compensate for their time.

During data quality control of the transcripts, NZ, AS, PL, and NM discovered that three interviews were conducted with individuals who did not meet the inclusion criteria for FSW and MSM. Two of these IDIs were with partners of people living with HIV, and one was with a man who did not identify as MSM. Upon verification and discussion, these three IDIs were excluded from the dataset, resulting in a final sample of 27 FSW and MSM.

### Quantitative Variables and Measurements

The primary outcome of interest was attrition from PrEP care, defined as missing scheduled appointments at one and three months after PrEP initiation. To reduce potential bias, we excluded repeat subjects (FSW and MSM who had initiated PrEP on a prior occasion) from the analysis. Additionally, the analysis of attrition at three months was limited to individuals who attended the one-month appointment.

Baseline sociodemographic data included age and marital status. Because the age distribution was skewed toward younger individuals, we categorized age into three groups: 18–30, 31–40, and over 40 years. Marital status was grouped into two categories: single versus married/cohabiting. Other covariates included the estimated number of sexual partners in the past six months (0–89 vs. 90+), categorized based on the median value of 90 partners due to a skewed, non-normal distribution; self-reported condom use in the past six months (used condoms regularly vs. not); sex work as the principal source of income in the past six months (yes vs. no); history of previous PrEP use (yes vs. no); and whether individuals had been diagnosed with an STI in the past six months (yes vs. no).

### Quantitative Data Analysis

First, we summarized programmatic data to create the PrEP cascade, detailing the numbers of individuals screened, found eligible, and initiated on PrEP. Next, we analyzed the retrospective cohort data, stratifying all analyses by key population (KP) groups—FSW and MSM—given their differing characteristics, health behaviors, and potential factors associated with PrEP use and attrition from care. Demographic and clinical characteristics of participants were summarized using counts and proportions. We calculated the proportion of participants retained at 1 and 3 months among those who initiated PrEP. Bivariate logistic regression models, including individuals with complete baseline data who initiated PrEP, were used to examine associations between baseline characteristics and attrition from care at months 1 and 3. Results were reported as odds ratios (ORs) with 95% confidence intervals (CIs) and p-values.

Multivariable models included baseline characteristics associated with the outcome variables at *p* < 0.05 or those considered clinically relevant based on prior literature. Associations were reported as adjusted odds ratios (aORs) with 95% CIs for attrition from PrEP care at 1 and 3 months. All analyses were conducted using SAS Statistical Analysis Software, version 9.4.

### Qualitative Data Analyses

IDIs were audio-recorded and professionally transcribed into text files in French or Lingala, according to the interview language. No participants declined audio recording. Transcripts in Lingala were then translated into French for analysis.

Data were analyzed using a combination of inductive and deductive approaches for thematic analysis. Our deductive approach was rooted in a socioecological model (SEM) [29], based on the hypothesis that multilevel factors—individual, interpersonal, institutional, and social—could impact PrEP engagement and experiences. The SEM guided the development of the IDI guide and the initial coding scheme.

The research team conducted readings of five transcripts, meeting regularly to identify and discuss common themes. Using this inductive approach, the team identified emerging themes and iteratively refined the codebook, resolving discrepancies by consensus. Four coders (AS, NM, NZ, and PL) used DeDoose, a web-based software for qualitative analysis [30], to apply codes to interview transcripts, with each transcript coded by at least two investigators. The team held regular meetings to review and discuss the coding process and resolve discrepancies as needed.

Upon completion of coding, we used Excel matrices to map identified themes according to the different levels of the SEM and to organize them by barriers and facilitators of PrEP use. Applying the same analytic principles, we also identified and examined differences by KP group (FSW vs. MSM) and by PrEP status (current users, stopped using, or never initiated PrEP).

### Data Integration and Synthesis

Integration in this multiple methods study occurred at several levels, including the conceptual underpinnings of the study design and planning, data analysis, interpretation, and reporting of results [31]. The integrated results were presented using a visual aid.

## Results

### Quantitative Results

**Fig. 1:**
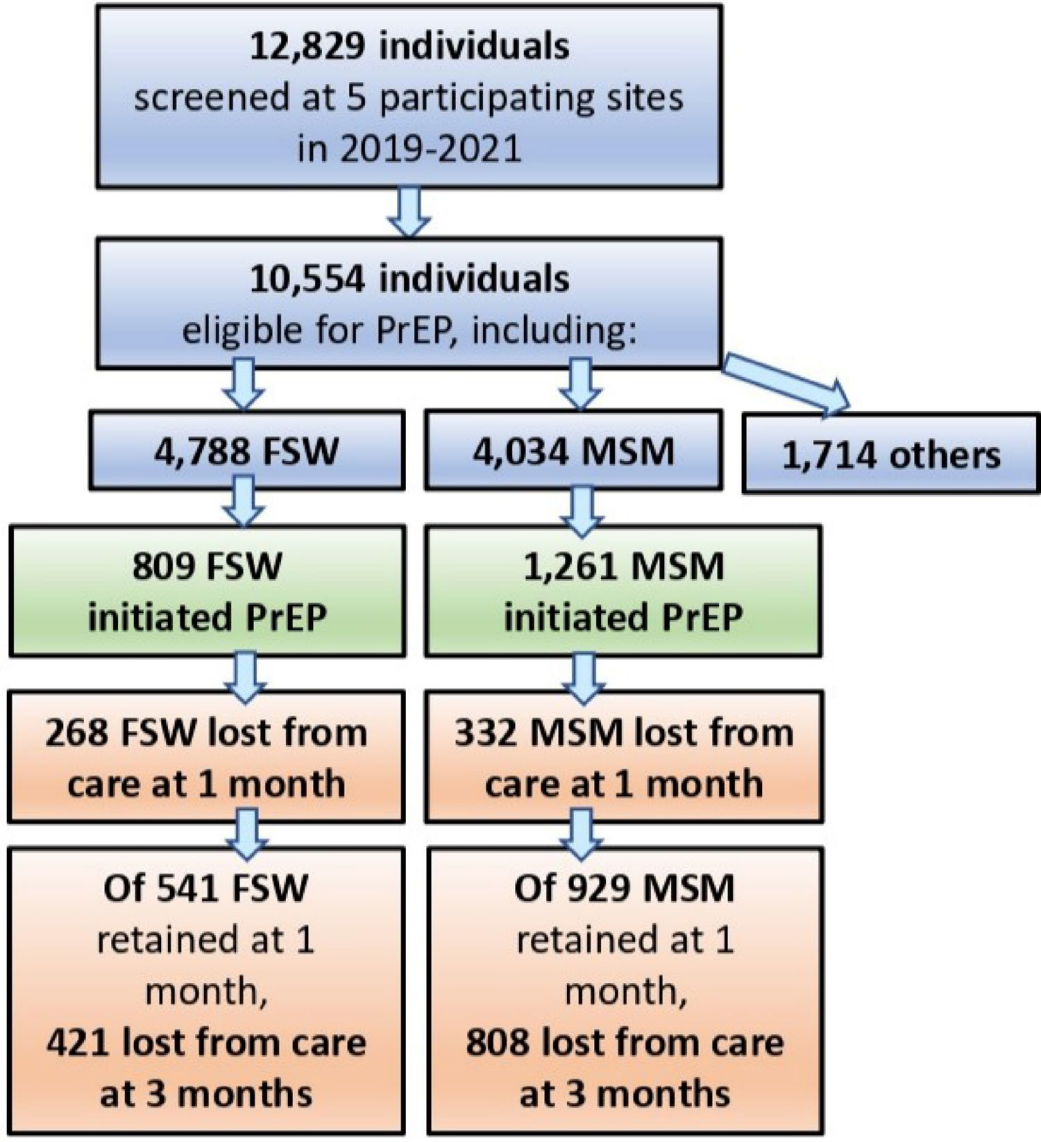
Flowchart of the study cohort

From 2019 to 2022, the five study sites cumulatively screened 12,829 people for PrEP. Of these, 10,554 (82%) were HIV-negative and eligible for PrEP: 4,788 (37%) FSW, 4,034 (31%) MSM, 1,703 (13%) clients of sex workers, and 11 (< 0.1%) HIV-negative partners of people living with HIV (PLWH). Out of 8,822 FSW and MSM who were screened and eligible for PrEP, 2,070 individuals (23.5%) initiated medication, including 809 (39%) FSW and 1,261 (61%) MSM (Fig. 1).

Among the 809 FSW who initiated PrEP, 804 (99.6%) were assigned female at birth (Table 1). The majority of FSW were aged 18–30 (69.9%, *n* = 561) and single (99.3%, *n* = 799). Most reported irregular condom use (64.8%, *n* = 520) and had over 90 sexual partners in the past six months (71%, *n* = 568). Sex work was the principal source of income for 91.4% of FSW (*n* = 732). The large majority (85.7%, *n* = 692) had not been diagnosed with STIs in the preceding six months, and 65.3% (*n* = 528) had not used PrEP previously.

**Table 1:** Socio-demographics characteristics of FSWs and MSM who initiated PrEP in 2019–2021

	FSW (*N* = 809), N (%)	MSM (*N* = 1,261), N (%)	Total (*N* = 2,070), N (%)
Sex at birth			
Male	3 (0.4)	1254 (99.4)	1257 (60.8)
Female	804 (99.6)	7 (0.6)	811 (39.2)
Age			
18–30	561 (69.9)	1018 (80.9)	1,579 (76.6)
31–40	194 (24.2)	187 (14.9)	381 (18.5)
> 40	48 (6.0)	53 (4.2)	101 (4.9)
Marital status			
Single/Divorced/Separated	799 (99.3)	1,255 (99.7)	2,054 (99.5)
Married/cohabiting	6 (0.7)	4 (0.3)	10 (0.5)
Number of sexual partners in the past 6 months			
0–89	232 (29.0)	803 (64.0)	1,035 (50.4)
90+	568 (71.0)	451 (36.0)	1,019 (49.6)
Regular condom use			
Yes	283 (35.2)	104 (8.3)	387 (18.8)
No	520 (64.8)	1150 (91.7)	1,670 (81.2)
Sex work being the principal source of income in the past 6 months			
Yes	732 (91.4)	271 (21.6)	1,003 (48.8)
No	69 (8.6)	985 (78.4)	1,054 (51.2)
Used PrEP before			
No	528 (65.3)	951 (75.4)	1,479 (71.4)
Yes	281 (34.7)	310 (24.6)	591 (28.6)
Had STIs in the past 6 months			
Yes	115 (14.3)	66 (5.3)	181 (8.8)
No	692 (85.7)	1179 (94.7)	1,871 (91.2)
Attrition from care by 1 month	268 (33.1)	332 (26.3)	600 (29)
Attrition from care by 3 months	421 (77.8)	808 (87)	1229 (83.6)

_* Frequencies may not add up due to missing data_

Attrition from PrEP care among FSW increased markedly by three months. At one month after initiation, 33.1% of FSW (*n* = 268) had not returned to study sites for scheduled appointments and PrEP refills. Of the 541 FSW who attended their one-month appointments, 77.8% (*n* = 421) had not returned for a three-month visit (Table 2). FSW who had not been diagnosed with an STI in the past six months were significantly less likely to be lost from care at one month post-initiation compared to those who reported having STIs (aOR 0.56, 95% CI 0.35–0.90, *p* < 0.05). By three months, older FSW (31–40 vs. 18–30; aOR 0.45, 95% CI 0.26–0.77, *p* < 0.01; >40 vs. 18–30; aOR 0.40, 95% CI 0.14–1.16, *p* < 0.1), those reporting a higher number of sexual partners (90 + vs. 0–89; aOR 0.37, 95% CI 0.17–0.81, *p* < 0.05), and those with previous experience using PrEP (yes vs. no; aOR 0.08, 95% CI 0.05–0.16, *p* < 0.001) were less likely to be lost from PrEP care.

**Table 2:** Factors associated with attrition from PrEP care by 1- and 3months among FSWs

	Attrition by 1 month (*n* = 268, 33.1%)			Attrition by 3 months (*n* = 421, 77.8%)		
	N	OR (95% CI)	aOR (95% CI)	N	OR (95% CI)	aOR (95% CI)
Age						
18–30	188			305		
31–40	55	0.79 (0.55, 1.12)	0.77 (0.53, 1.11)	96	0.50 (0.32, 0.78)**	0.45 (0.26, 0.77)**
> 40	22	1.68 (0.93, 3.04)^+^	1.51 (0.82, 2.78)	17	0.42 (0.18, 0.98)*	0.40 (0.14, 1.16) ^+^
Number of sexual partners in the past 6 months						
0–89	69			151		
90+	196	1.24 (0.89, 1.73)	1.31 (0.89, 1.93)	265	0.20 (0.10, 0.37)***	0.37 (0.17, 0.81)*
Regular condom use						
Yes	99			139		
No	166	0.87 (0.64, 1.18)	1.20 (0.81, 1.78)	279	1.20 (0.79, 1.84)	1.68 (0.93, 3.03) ^+^
Sex work principal source of income in the past 6 months						
Yes	239			399		
No	23	1.03 (0.61, 1.74)	1.05 (0.60, 1.82)	21	0.20 (0.11, 0.37)***	0.27 (0.12, 0.60)***
Used PrEP before						
Yes	187			317		
No	81	0.74 (0.54, 1.01)^+^	0.71 (0.49, 1.01)^+^	104	0.08 (0.05, 0.14)***	0.08 (0.05, 0.16)***
Had STIs in the past 6 months						
Yes	53			52		
No	214	0.52 (0.35, 0.78)**	0.56 (0.35, 0.90)*	368	0.64 (0.32, 1.31)	1.39 (0.57, 3.41)

+ *p*<0.1; * *p* <0.05; ** *p*<0.01; *** *p*<0.001

Of the 1,261 MSM who initiated PrEP, 1,254 (99.4%) were assigned male at birth. The majority were young (80.9% aged 18–30, *n* = 1,018) and single (99.7%, *n* = 1,255). Most MSM reported irregular condom use (91.7%, *n* = 1,150) and having 0–89 sexual partners in the past six months (64%, *n* = 803). For 21.6% of MSM (*n* = 271), sex work was the principal source of income. The majority (94.7%, *n* = 1,179) had not been diagnosed with STIs in the preceding six months and had not used PrEP previously (75.4%, *n* = 951). Similar to FSW, attrition from PrEP care among MSM was high: 26.3% (*n* = 332) did not return to study sites at one month after PrEP initiation. Of the 929 MSM who attended their one-month appointments, 87% (*n* = 808) had not returned for a three-month visit (Table 3).

**Table 3:** Factors associated with attrition from PrEP care by 1- and 3months among MSM

	Attrition by 1 month (*n* = 332, 26.3%)			Attrition by 3 months (*n* = 808, 87%)		
	N	OR (95% CI)	aOR (95% CI)	N	OR (95% CI)	aOR (95% CI)
Age						
18–30	273			654		
31–40	43	0.81 (0.56, 1.18)	0.79 (0.54, 1.16)	120	0.70 (0.43, 1.14)	0.88 (0.50, 1.54)
> 40	15	1.08 (0.58, 1.99)	1.03 (0.53, 1.98)	32	0.74 (0.30, 1.82)	1.27 (0.41, 3.92)
Number of sexual partners in the past 6 months						
0–89	189			546		
90+	142	1.49 (1.15, 1.93)**	1.29 (0.98, 1.70) ^+^	257	0.62 (0.42, 0.91)*	0.72 (0.46, 1.12)
Regular condom use						
Yes	33			54		
No	298	0.75 (0.49, 1.16)	0.74 (0.45, 1.21)	749	2.29 (1.28, 4.10)**	0.72 (0.30, 1.71)
Sex work principal source of income in the past 6 months						
Yes	74			166		
No	258	0.94 (0.70, 1.28)	1.16 (0.84, 1.61)	638	1.34 (0.86, 2.09)	1.18 (0.73, 1.91)
Used PrEP before						
Yes	193			684		
No	139	3.19 (2.43, 4.20)***	3.59 (2.69, 4.79)***	124	0.29 (0.19, 0.43)***	0.25 (0.16, 0.39)***
Had STIs in the past 6 months						
Yes	30			24		
No	302	0.41 (0.25, 0.68)***	0.33 (0.19, 0.57)***	780	4.02 (1.95, 8.30)***	4.56 (2.00,10.40)***

+ *p*<0.1; * *p* <0.05; ** *p*<0.01; *** *p*<0.001

MSM who had not been diagnosed with an STI in the past six months were significantly less likely to be lost from care at one month compared to those who reported having STIs (aOR 0.33, 95% CI 0.19–0.57, *p* < 0.001). A higher number of sexual partners (90 + vs. 0–89; aOR 1.29, 95% CI 0.98–1.7, *p* < 0.1) and no previous experience using PrEP (no vs. yes; aOR 3.59, 95% CI 2.69–4.79, *p* < 0.001) were associated with attrition from care at one month. At three months, the pattern of attrition reversed: the absence of a diagnosed STI (no vs. yes; aOR 4.56, 95% CI 2.00–10.40, *p* < 0.001) was associated with increased attrition, whereas prior experience using PrEP (yes vs. no; aOR 0.25, 95% CI 0.16–0.39, *p* < 0.001) was associated with a lower likelihood of attrition.

### Qualitative Results

From November 2021 to March 2022, 27 in-depth interviews were conducted with participants aged 22 to 52 years, including 14 female sex workers (FSW) and 13 men who have sex with men (MSM). At the time of the interviews, 14 participants were current PrEP users, 6 were former users, and 7 had never used PrEP. Most participants had completed secondary education (63%, *n* = 17) and were single, divorced, or separated (85%, *n* = 23) (Table 4). All FSW reported exchanging sex for money, with two-thirds (*n* = 9) having 400–600 clients in the previous six months, and 70% (*n* = 10) reporting a diagnosed STI during that period. Among MSM, 46% (*n* = 6) exchanged sex for money in the past six months, 73% (*n* = 8) had a recent STI, and 82% (*n* = 9) reported 1 to 20 sexual partners in that timeframe.

**Table 4:** Characteristics of IDI participants (*n* = 27)

	FSW, *N* (%)	MSM, *N* (%)
Age		
18–30	6 (43)	8 (62)
31–40	3 (21)	2 (15)
> 40	5 (36)	3 (23)
Marital status		
Single/Divorced/Separated	11 (79)	12 (92)
Married/cohabiting	3 (21)	1 (8)
Education		
Primary school	2 (14)	1 (8)
Secondary school	10 (72)	7 (54)
Some university	2 (14)	5 (38)
PrEP use		
Current	7 (50)	7 (54)
Prior	3 (21)	3 (23)
Never	4 (28)	3 (23)
Sex at birth		
Female	12 (86)	0
Male	2 (14)	13 (100)
Gender identity		
Female	14 (100)	0
Male	0	11 (85)
Gender fluid	0	2 (15)
Sexual partners		
Men	14 (100)	6 (46)
Men and women	0	7 (54)
Number of sexual partners in the past 6 months		
1–20	3 (21)	9 (82)
20–99	1 (15)	1 (9)
> 100	9 (64)	1 (9)
Had STIs in the past 6 months	10 (71)	8 (73)
Exchanged sex for money in the past 6 months	14 (100)	6 (46)
Reported challenges related to condom use		
No challenges	4 (33)	2 (18)
Condoms feel uncomfortable and/or reduce pleasure	2 (17)	6 (55)
Condoms can tear	4 (33)	0
Clients or partners unwilling to use condoms	2 (17)	3 (27)
Time since most recent HIV test		
< 1 month	4 (33)	2 (15)
2–5 months	7 (59)	7 (54)
> 6 months	1 (8)	4 (31)

Our analysis revealed several emergent themes. Knowledge about PrEP as a means of HIV prevention was high, and all participants were aware that PrEP was available at KP-friendly health facilities and drop-in centers. Healthcare providers and social networks served as the main sources of information, encouragement, and support for PrEP use.

Despite these important facilitators, significant barriers often outweighed perceived benefits of PrEP. Concerns that discouraged FSW and MSM from initiating or consistently using PrEP included individual-level factors such as side effects and pill burden, multiple forms of stigma operating at different levels, and logistical challenges such as the distance to health centers and inconsistent availability of PrEP.

#### Side Effects and Pill Burden

Side effects of PrEP emerged as a significant barrier to medication use. Anticipated side effects—often influenced by peer conversations and shared experiences—discouraged some participants from initiating PrEP. In particular, participants who heard about side effects from others or witnessed them first-hand were more hesitant to begin treatment. For example, 2 out of 7 participants (29%) who had never used PrEP reported that they had heard negative things about its side effects.

The majority of FSW and MSM who were either currently using PrEP or had discontinued the medication (90%, *n* = 18) reported experiencing a range of side effects. The most commonly cited were fatigue and dizziness, followed by nausea, headaches, heartburn, and increased salivation and urination.

PrEP makes me really dizzy—it’s like the earth is spinning quickly beneath my feet. I also experienced headaches in the beginning.

*Participant 26*,* a 32-year-old female*,* is currently using PrEP*.

Side effects were the primary reason for discontinuation of PrEP among both FSW and MSM (83%, n = 5). Pill burden—often described as “having had enough” or “having too much” PrEP—compounded the impact of side effects. These factors were most frequently cited as reasons for stopping PrEP use:

*“I felt that taking [PrEP] every day was becoming a burden. I was constantly tired*,* and sometimes I had stomach aches—eh! I talked about it with him [the doctor] early on; I asked to stop*,* but he reassured me*,* saying*,* “Keep taking it!” Still*,* I couldn’t continue—I gave up”.*

*Participant 18*,* a 42-year-old female*,* previously used PrEP*

I stopped because I realized that I had consumed a lot. Sometimes, I experienced heartburn! That’s why I stopped.

*Participant 1*,* a 47-year-old female*,* previously used PrEP*.

The large size of the pill was another concern for some participants (n = 3), which further contributed to their dislike of oral PrEP:

*“I stopped because of side effects that developed—nausea and vomiting—and the size of the pills. The pills are big*,* really big. When you take them*,* it feels like something is stuck in your throat. Eventually*,* my heart couldn’t handle it anymore.*

*Participant 8*,* a 25-year-old male*,* previously used PrEP*.

Several FSW (*n* = 3) reported that experienced side effects, primarily fatigue and dizziness, made performing sex work more difficult. Since sex work was their main source of income, they sought solutions such as moving PrEP intake to the morning to have more energy at night, or ultimately abandoning the medication. Alcohol use was also an important part of the lifestyle for both FSW and MSM, who might drink with clients or socialize in bars and other venues frequented by KPs. During PrEP consultations, healthcare providers either advised against alcohol use while on PrEP or did not provide information about the absence of known interactions between PrEP and alcohol. Two MSM participants and one FSW expressed concerns about their ability to drink while using PrEP, which complicated their adherence to the medication.

Side effects, pill burden, challenges integrating PrEP into daily routines, and other factors were intertwined with a broader societal and cultural dislike of medications. Although participants were initially interested in PrEP’s effective protection against HIV, many grew wary of taking a daily pill and often questioned, “Why take medication if I’m not sick?” A large proportion of participants (44%, *n* = 12), regardless of their PrEP status, expressed a general dislike of taking medications, especially on a daily basis. Among those who had never used PrEP, both FSW and MSM often did not specify their concerns clearly, offering vague explanations such as “this is not for me,” “not now, but I may try one day,” or similar statements. One FSW who never initiated PrEP gave a succinct explanation of her reluctance, framing it strongly in terms of “addiction”:

“*I don’t like something that demands so much from me. I mean*,* that I get addicted to* it—taking it every evening—because they [doctors] say it must be taken daily, and I don’t like taking [medical] products every day.”

*Participant 27*,* a 23-year-old female*,* never used PrEP*.

#### HIV and PrEP Stigmas

The majority of participants (78%, *n* = 21) reported experiencing or anticipating various forms of PrEP-related stigma. Because PrEP bottles closely resemble those used for antiretroviral treatment (ART) for HIV, many participants faced questions and rumors from family, friends, and significant others who confused PrEP with ART. The daily use of medication was often assumed to be treatment for illness rather than prevention. Due to widespread stigma and this confusion between PrEP and ART, participants preferred to take PrEP privately at home to maintain confidentiality, and many FSW and MSM chose not to disclose their PrEP use to anyone. For some, anticipated stigma was a barrier that prevented them from initiating PrEP altogether:

*“One difficulty is that if you take PrEP in public*,* people will think you have HIV”.*

*Participant 10*,* a 47-year-old male*,* never used PrEP*

I refused to take PrEP because of the packaging; the bottle, essentially—the same bottle as ART.

*Participant 21*,* a 27-year-old male*,* never used PrEP*.

In the context of powerful stigmas operating at individual, relational, and societal levels, hesitancy about using PrEP and decisions regarding its initiation or discontinuation were often rooted in the perceived balance between benefits and negative outcomes, as well as the dynamics of relationships with others. Participants who prioritized their health and protection against HIV, like this participant, adhered to PrEP despite stigma:

People were saying, “He takes medicine for people with AIDS!” but I didn’t care much.

*Participant 3*,* a 23-year-old male*,* is currently using PrEP*.

Other participants struggled and, despite recognizing PrEP as an effective form of protection, ultimately discontinued its use due to side effects and stigma occurring at multiple levels:

First of all, I was ashamed because I constantly heard from others that the medicine we take is the same as what sick people [with HIV] take. I felt ashamed even at home; my family suspected me. They thought I wasn’t clean, that when you start taking big tablets like that, you must have HIV or something like that. But I held on. Even when I came to the center to take my medicine, you could see how they looked at me. That’s what bothered me. It was my goal, so I continued, but eventually, I stopped.

*Participant 8*,* a 25-year-old male*,* previously used PrEP*.

Most participants (70%, *n* = 19) acknowledged a lack of information or misinformation about PrEP among key populations and the broader Congolese society, which contributed to stigma surrounding PrEP use. Participants noted that this lack of awareness caused doubts about PrEP’s effectiveness, reinforced negative labeling of PrEP users, and perpetuated HIV stigma. They suggested that raising awareness about PrEP could improve its acceptance in society and potentially reduce stigma.

#### Limited number of KP-friendly facilities and PrEP availability

Finally, logistical barriers—including the limited availability of PrEP at KP-friendly health centers and DICs—were important obstacles to PrEP engagement among FSW and MSM. Nearly a third of participants (29%, *n* = 8) mentioned the challenge of long distances between their homes and facilities offering PrEP services. With an estimated population of 17 million, Kinshasa is the most populous city in the DRC, and significant traffic congestion combined with a lack of public transportation make traveling across the city complicated and time-consuming.

One difficulty people may encounter is the distance; the journey to the center is quite long.

*Participant 19*,* a 27-year-old female*,* never used PrEP*.

Combined with potential PrEP stockouts at health facilities and DICs, accessing PrEP services and medication availability may pose challenges that affect KPs’ decisions to use PrEP consistently. FSW and MSM living closer to study sites frequented them more often, while those living farther away had to navigate long distances and heavy traffic. Participants felt that both themselves and KP communities overall would benefit from easier access to PrEP services and expressed a strong preference for establishing more centers offering PrEP across the city.

I think we need to set up several centers in the city. That will make it easier for everyone to access [PrEP] from their homes.

*Participant 10*,* a 47-year-old male*,* never used PrEP*.

### Integration of Quantitative and Qualitative Results

The quantitative and qualitative components of this study highlight the limited utilization of effective HIV prevention offered by PrEP among FSW and MSM in the DRC. Between 2019 and 2021, only one-quarter of FSW and MSM who were screened at participating sites and deemed eligible for PrEP actually initiated it (Fig. 1; Source 1). Significant dropouts along the PrEP care cascade underscore high attrition rates. Of the 2,070 FSW and MSM who initiated PrEP, 29% did not return for scheduled clinical visits and medication refills at 1 month. By 3 months post-initiation, among those who attended their 1-month visits, 78% of FSW and 87% of MSM had not returned for their 3-month appointments.

Qualitative findings identified key barriers that may explain the low uptake of PrEP and high attrition among Congolese FSW and MSM. Experienced and anticipated side effects, pill burden, and challenges related to daily pill intake were important individual-level barriers, often leading to PrEP discontinuation. Stigma associated with PrEP—primarily due to its similarity in appearance to ART—was another significant barrier. PrEP users faced suspicion and judgment from family, friends, and peers, which complicated efforts to maintain confidentiality. This confusion between PrEP and ART fueled fears and misconceptions about participants’ HIV status within their social circles. Even those who recognized PrEP’s efficacy struggled to continue use due to combined stigma and pressure from family, peers, and their communities.

Additionally, structural barriers such as the limited number of low-stigma, KP-friendly facilities offering PrEP in Kinshasa, long distances to these sites, and periodic stockouts further impeded access to PrEP. These individual, interpersonal, and institutional challenges help explain the underutilization of PrEP among Congolese FSW and MSM.

## Discussion

Findings from this study align with evidence from other African settings, which show that despite high retention rates observed in demonstration projects, attrition from PrEP care in real-world contexts remains high. We observed that attrition at 1 and 3 months among Congolese FSW and MSM was substantial, and possibly higher than reported in other sub-Saharan African settings. For instance, programmatic data from Cameroon indicated attrition rates of 63% at 3 months, 72% at 6 months, and 81% at 12 months among FSW and MSM [18]. A systematic review of PrEP use among FSW in SSA found that 34% discontinued PrEP care by 6 months [16]. Evidence on attrition among African MSM is mixed; a Kenyan study reported 30% lost to follow-up by 6 months [15], whereas in Benin, only 12% were lost [32]. In Kenya, residence outside the clinic’s immediate catchment area was independently associated with loss to follow-up among MSM [15]. This finding supports our qualitative results, which suggest that long distances and travel times to KP-friendly facilities may be significant barriers for some FSW and MSM.

We also hypothesize that the higher attrition from care observed in the DRC, compared to other settings, may be partially attributed to language barriers and the training of healthcare professionals. While English-speaking countries in sub-Saharan Africa—such as Kenya, Rwanda, South Africa, and Uganda—have received considerable international attention for HIV research and implementation strategies, French-speaking regions have not received the same attention [33, 34]. This disparity may contribute to the lack of targeted efforts to develop and implement training programs in French for healthcare providers, create PrEP informational materials in French and local languages, and launch other supportive initiatives.

High attrition from PrEP care in the DRC should be understood within the broader context of patterns of PrEP discontinuation and re-initiation observed in other sub-Saharan African and global settings. In our retrospective cohort of 2,070 FSW and MSM who initiated PrEP (Fig. 1; Source 2), nearly one-third had prior experience using PrEP. A systematic review found that globally, 24% of people who had stopped PrEP later restarted the medication, with higher rates of re-initiation reported in African studies compared to those from the USA [35]. Similarly, a prospective cohort study of MSM and transgender women in Kenya showed that over a 20-month follow-up period, 40% of participants stopped PrEP, and among them, 51% restarted [36]. These findings from diverse contexts suggest that individuals may require multiple attempts before fully engaging with PrEP. Moreover, patterns of PrEP use may vary according to changes in HIV exposure risk, highlighting the need for flexibility to stop and restart PrEP as clinically appropriate. Importantly, removal of barriers to PrEP access has been identified as a key factor motivating re-initiation [35].

Targeting the major barriers to PrEP engagement identified in our study may help increase PrEP utilization and reduce new HIV infections among Congolese FSW and MSM. The high rates of prior PrEP experience—suggestive of patterns of stopping and restarting—further underscore the need for strategies that address these barriers and improve access, ultimately supporting more consistent use of the medication. Consistent with global evidence identifying PrEP-related stigma as a significant, multi-level barrier to use [37–44], our study found stigma to be a critical obstacle to PrEP engagement. Additionally, a culturally rooted dislike of daily medication use outside of illness contexts emerged as another key barrier. Previous research has emphasized the influence of cultural factors on concerns about daily medication intake, which can affect adherence, including to antiretroviral treatment for HIV [44, 45]. Although limited data exist on cultural attitudes toward daily oral PrEP in African settings, our findings suggest an urgent need for strategies that address beliefs surrounding medication use in the absence of illness.

Activities to raise awareness among both the general Congolese population and key populations are strongly recommended to prevent the stigmatization of PrEP users and the perception that they are living with HIV. Healthcare providers, peer educators, and the social networks of FSW and MSM can play a vital role in awareness-raising efforts. Evidence from the DRC and Rwanda highlights the importance of healthcare professionals in building rapport with KPs during clinical visits and providing information that supports both PrEP uptake and persistence [17, 20]. Studies from Nigeria and Zimbabwe have shown that KPs support the introduction of PrEP services in familiar settings, such as health facilities and drop-in centers, and value the encouragement and support offered by clinical staff and peers. These combined factors may help counteract stigma and alleviate fears related to side effects [40, 46]. Strengthened messaging from Congolese healthcare providers and peer educators can promote sustained engagement with PrEP, especially in light of high rates of discontinuation and re-initiation. Additionally, implementing new or refresher training programs in French for providers and peer educators involved in PrEP service delivery is recommended.

We found that FSW and MSM valued the PrEP services provided at the participating sites. However, the number of KP-friendly facilities in the large city of Kinshasa is very limited, creating logistical challenges in accessing PrEP services. To improve PrEP access and availability, the number of facilities offering care in a non-stigmatizing environment for vulnerable populations must be significantly increased. Community-based organizations (CBOs) that serve FSW, MSM, and LGBTQ + individuals—and are located in various parts of Kinshasa and throughout the DRC—represent promising platforms for low-threshold PrEP service delivery. Although our study included two CBOs, expanding this network to other areas could benefit individuals at high risk for HIV and help prevent new infections.

Finally, long-acting injectable (LAI) PrEP has the potential to address overlapping HIV and PrEP-related stigma, as well as challenges associated with daily pill-taking. Although LAI PrEP has not yet been registered or introduced in the DRC, its potential to reduce stigma, alleviate pill fatigue, and lower attrition from care—along with a likely high demand among Congolese FSW and MSM—underscores the need for policies supporting its introduction in the country.

Several limitations of this study should be acknowledged. The study was conducted in Kinshasa, the capital of the DRC, and participants recruited from this urban setting may not reflect the experiences of key populations in rural areas. Due to the nature and completeness of data extracted from clinical registries of PrEP users, only a limited number of variables were assessed, which may restrict our understanding of factors associated with attrition from care. Additionally, data on PrEP adherence were not collected. Willingness to participate in in-depth interviews may have been influenced by social desirability bias; some individuals may have been too stigmatized to take part in research about PrEP or may have been reluctant to share their experiences during interviews conducted at healthcare facilities or DICs. As a result, we may not have captured the perspectives of key population members who had negative experiences accessing PrEP services in clinical settings.

## Conclusions

**Fig. 2:**
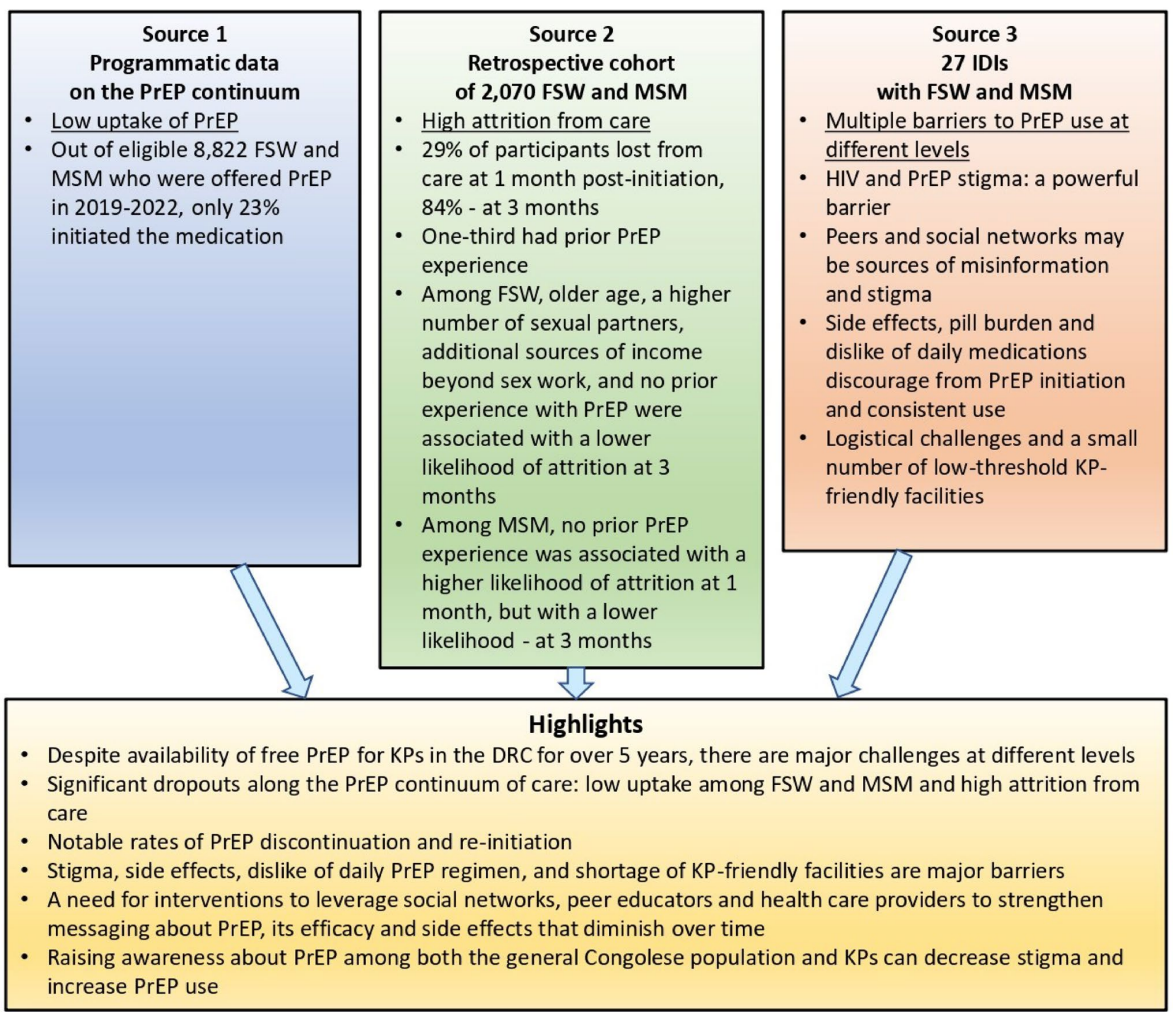
Joint display of the study findings

Findings from this multiple methods study revealed low rates of PrEP initiation and high attrition from care among Congolese FSW and MSM (see Fig. 2). Despite PrEP being available in the DRC for over five years, significant barriers remain that hinder its uptake among key populations: stigma surrounding PrEP, side effects, aversion to the daily PrEP regimen, and a shortage of KP-friendly facilities offering the service. Targeted interventions that engage KP social networks, peer educators, and healthcare providers are recommended to strengthen messaging about PrEP, its effectiveness, and the typically temporary nature of side effects. Raising awareness about PrEP among both the general Congolese population and key populations can help reduce stigma and improve engagement. Expanding the number of low-threshold facilities offering PrEP services can further enhance access for key populations at risk and help prevent new HIV infections.
